# Supplementary figures and images for: WE economy: Potential of mutual aid distribution based on moral responsibility and risk vulnerability
Source: PLoS One. 2024 May 16;19(5):e0301928. doi: 10.1371/journal.pone.0301928 (PMC11098405; doi:10.1371/journal.pone.0301928)

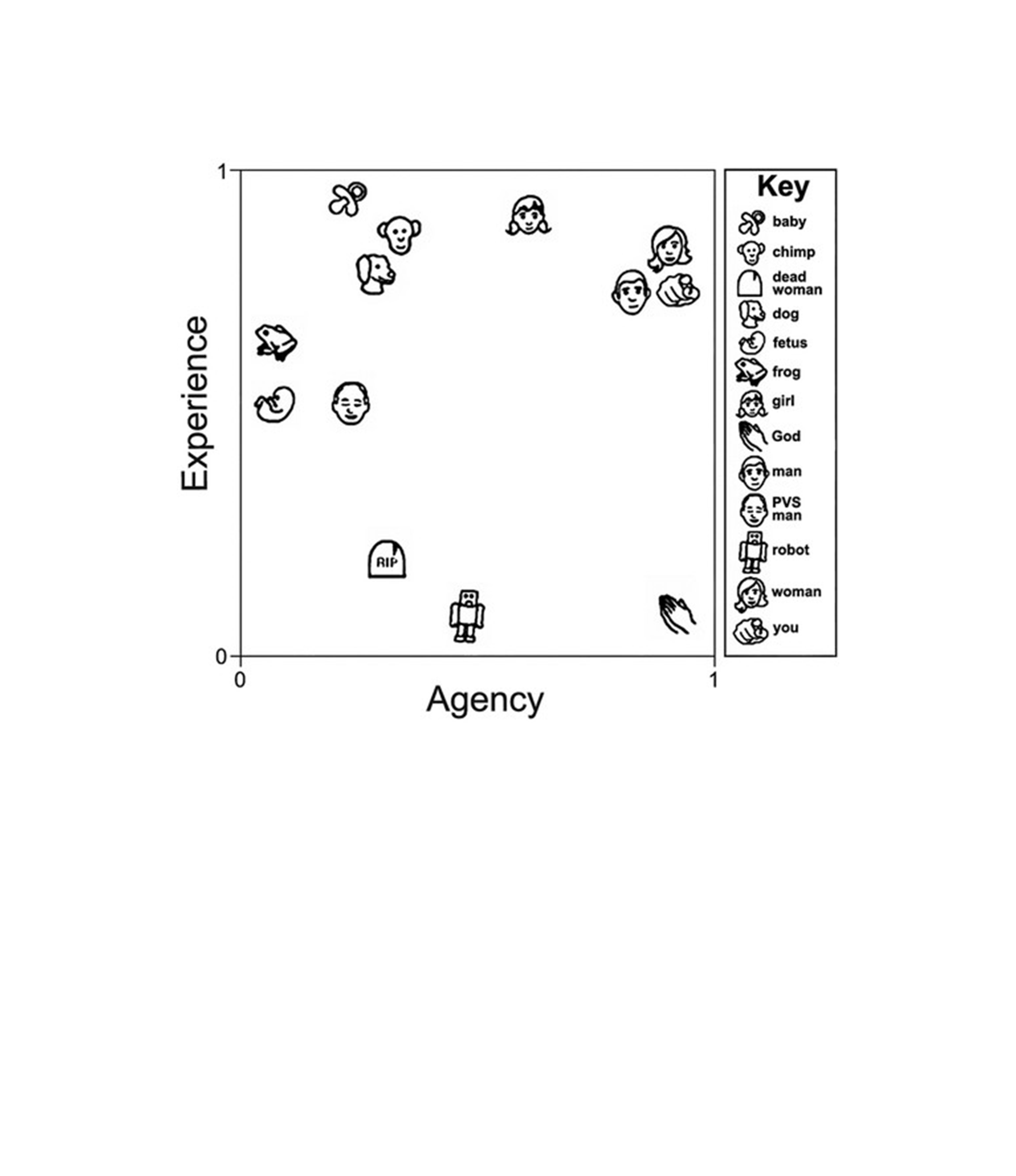

Supplement: S1 Fig — (Gray, Gray, and Wegner 2007 [15]) Copyright Clearance Center’s RightsLink® License Number: 5695221129353. (TIF) [file pone.0301928.s001.tif]
