# Supplementary material for: WE economy: Potential of mutual aid distribution based on moral responsibility and risk vulnerability
Source: PLoS One. 2024 May 16;19(5):e0301928. doi: 10.1371/journal.pone.0301928 (PMC11098405; doi:10.1371/journal.pone.0301928)
Supplement: Attachment — Submitted filename: Comments to the Author.docx [file pone.0301928.s003.docx]

The manuscript offers an innovative approach to wealth distribution through the introduction and analysis of three models. However, it could benefit from a more robust data and methodology section, ensuring the reliability of the simulation results and the representativeness of the data used. The empirical section requires further enrichment, possibly through the inclusion of diverse datasets, case studies, or field experiments to validate the models and assess their performance in various economic contexts. Addressing these aspects would significantly enhance the paper's rigour, practical applicability, and contribution to the field.

1. Addressing these major issues is crucial for enhancing the manuscript's academic rigor and practical relevance. Integrating a new joint model that encapsulates the essence of moral responsibility and risk vulnerability fills a notable gap in the existing literature. Ensuring this integration is articulated and substantiated effectively will solidify the paper's contribution and establish its necessity in advancing the discourse on wealth distribution models.
2. The manuscript did not explicitly discuss the significance of constructing the joint model. It focused more on introducing and comparing individual models based on moral responsibility and risk vulnerability. Highlighting the significance of integrating these models could provide a more comprehensive understanding of their collective potential and applicability in addressing wealth inequality.
3. The new joint model's marginal contribution lies in addressing gaps left by previous models. It's essential as it potentially offers a more holistic and integrated approach to wealth distribution, tackling issues related to moral responsibility and risk vulnerability more effectively. By considering these factors, the joint model could provide a more comprehensive and nuanced solution to wealth inequality, addressing complexities that individual models may not fully capture.
4. The introduction should more explicitly identify the specific research gap being addressed.
5. The study's aims should be distinctly stated within the framework of the theoretical background introduced.
6. The introduction should better articulate how current models inadequately address moral responsibility and risk vulnerability, justifying the new "WE economy" model.
7. The methodology should include a sensitivity analysis to demonstrate the model's robustness against parameter variations.
8. The discussion should engage with the practical challenges and propose solutions for actualizing the WE economy model in real-world scenarios.
