# Supplementary material for: WE economy: Potential of mutual aid distribution based on moral responsibility and risk vulnerability
Source: PLoS One. 2024 May 16;19(5):e0301928. doi: 10.1371/journal.pone.0301928 (PMC11098405; doi:10.1371/journal.pone.0301928)
Supplement: Attachment — Submitted filename: Response_to_Reviewers_0322_2.docx [file pone.0301928.s004.docx]

March 22, 2024

Baogui Xin

Academic Editor

*PLOS ONE*

Dear Editor and Reviewers:

Thank you for your suggestions and comments on our manuscript titled “WE economy: Potential of mutual aid distribution based on moral responsibility and risk vulnerability.” I also appreciate the time and effort you and each of the reviewers have dedicated to providing insightful feedback on ways to strengthen our paper. I have incorporated changes that reflect the detailed suggestions you have so graciously provided. I hope that my edits and the responses provided below satisfactorily address all the issues and concerns you and the reviewers have noted.

To facilitate your review of my revisions, the following is a point-by-point response to the questions and comments delivered in your letter [PONE-D-23-43602].

**Response to Editor:**

1. Recommend that it should be revised taking into account the changes requested by the reviewers.

**Response:** Thank you for the recommendation. I revised the manuscript, considering the reviewers’ suggestions.

2. Include the following items when submitting your revised manuscript: ‘Response to Reviewers,’ ‘Revised Manuscript with Track Changes,’ and ‘Manuscript’ without tracked changes.

**Response:** Following your instruction, I have submitted three items.

**Response to Journal Requirements:**

1. Ensure that your manuscript meets *PLOS ONE*’s style requirements.

**Response:** I have checked the style requirements.

2. Note that *PLOS ONE* has specific guidelines on code sharing for submissions.

**Response:** I have shared the code, following best practices.

3. State what role the funders took in the study. Include this amended Role of Funder statement in your cover letter.

**Response:** I have included the Role of Funder statement in the cover letter.

4. Expand the acronym “JSPS” (as indicated in your financial disclosure) so that it states the name of your funders in full. This information should be included in your cover letter.

**Response:** I have stated the names of my funders in full in the cover letter.

5. Funding information should not appear in the Acknowledgments section or other areas of your manuscript. Include your amended statements within your cover letter.

**Response:** I have removed the funding information from the Acknowledgments section and included it in the cover letter.

6. Confirm at this time whether or not your submission contains all raw data required to replicate the results of your study.

**Response:** I have provided all the data underlying the findings in this study.

7. Review your reference list to ensure that it is complete and correct.

**Response:** I have reconfirmed that the reference list is correct.

**Response to Reviewer #1:**

1. It could benefit from a more robust data and methodology section, ensuring the reliability of the simulation results and the representativeness of the data used. The empirical section requires further enrichment, possibly through the inclusion of diverse datasets, case studies, or field experiments to validate the models and assess their performance in various economic contexts. Addressing these aspects would significantly enhance the paper’s rigour, practical applicability, and contribution to the field.

**Response:** Thank you for your helpful suggestions. The proposal for a new joint model in this study did not include a case study of itself, but to follow your advice, I have cited the existing literature and figure in the Results section as very helpful data and added a comparison between them and the new joint model (Fig 9 and reference [36], pp. 22–23, lines 485–518). Furthermore, I have mentioned them in the Discussion section (p. 24, lines 534, 535, and 538), and finally, I have added the need for empirical research through case studies as well as fieldwork as future challenges (p. 29, line 657). Note that the page and line numbers are from ‘Revised Manuscript with Track Changes.’

2. Integrating a new joint model that encapsulates the essence of moral responsibility and risk vulnerability fills a notable gap in the existing literature. Ensuring this integration is articulated and substantiated effectively will solidify the paper’s contribution and establish its necessity in advancing the discourse on wealth distribution models.

**Response:** I have added the significance of the joint model as in #3 below and the research gap as in #4 below.

3. The manuscript did not explicitly discuss the significance of constructing the joint model. It focused more on introducing and comparing individual models based on moral responsibility and risk vulnerability. Highlighting the significance of integrating these models could provide a more comprehensive understanding of their collective potential and applicability in addressing wealth inequality.

**Response:** I have added the significance and importance of a joint model encapsulating moral responsibility and risk vulnerability (p. 6, lines 113–115, p. 19, lines 406–409, p. 25, lines 550–551).

4. The new joint model’s marginal contribution lies in addressing gaps left by previous models. It's essential as it potentially offers a more holistic and integrated approach to wealth distribution, tackling issues related to moral responsibility and risk vulnerability more effectively. By considering these factors, the joint model could provide a more comprehensive and nuanced solution to wealth inequality, addressing complexities that individual models may not fully capture.

**Response:** I have added a description of the research gap (p. 6, lines 116­–126, p. 7, lines 147–152) and references [18–20].

5. The introduction should more explicitly identify the specific research gap being addressed.

**Response:** As written in response to #4 above, I have added the research gap.

6. The study’s aims should be distinctly stated within the framework of the theoretical background introduced.

**Response:** I have revised and added the study’s aim based on the research gap with the existing literature (p. 7, lines 155–158, lines 161–162).

7. The introduction should better articulate how current models inadequately address moral responsibility and risk vulnerability, justifying the new “WE economy” model.

**Response:** As written in response to #4 above, I have added the inadequacies of the current models.

8. The methodology should include a sensitivity analysis to demonstrate the model’s robustness against parameter variations.

**Response:** I have added the calculation results for parameter variations (Figs 7 and 8, pp. 20–22, lines 429–484).

9. The discussion should engage with the practical challenges and propose solutions for actualizing the WE economy model in real-world scenarios.

**Response:** I have added a note on practical challenges (pp. 27–28, lines 613–625, and references [48,49]).

**Response to Reviewer #2:**

1. P. 7, line 153: The notation used for risk vulnerability may be incorrect. Please review and confirm.

**Response:** Thank you for pointing this out. I have corrected the risk vulnerability symbol from $\rho_{Mi}$ to $\rho_{Ri}$ (p. 8, line 193).

2. P. 9, lines 175–176: The subscript “k” is introduced without a clear definition. Please clarify what “k” represents.

**Response:** I have added the meaning of the subscript “k” (p. 10, lines 219–220).

Thank you once again for giving me the opportunity to strengthen my manuscript with your valuable comments and queries. I have worked hard to incorporate your feedback and hope that these revisions persuade you to accept our submission.

Sincerely,

Takeshi Kato

Hitachi Kyoto University Laboratory, Open Innovation Institute, Kyoto University

Yoshidahonmachi, Sakyo-ku, Kyoto-shi, Kyoto 606-8501, Japan

+81-75-753-9716

kato.takeshi.3u@kyoto-u.ac.jp
